# Acute Gastroenteritis Surveillance through the National Outbreak Reporting System, United States

**DOI:** 10.3201/eid1908.130482

**Published:** 2013-08

**Authors:** Aron J. Hall, Mary E. Wikswo, Karunya Manikonda, Virginia A. Roberts, Jonathan S. Yoder, L. Hannah Gould

**Affiliations:** Centers for Disease Control and Prevention, Atlanta, Georgia, USA

**Keywords:** acute gastroenteritis, norovirus, outbreaks, surveillance, foodborne disease, waterborne disease, United States, viruses, bacteria, parasites, toxins, chemicals, non-infectious causes, pathogens, enteric infections

## Abstract

Implemented in 2009, the National Outbreak Reporting System provides surveillance for acute gastroenteritis outbreaks in the United States resulting from any transmission mode. Data from the first 2 years of surveillance highlight the predominant role of norovirus. The pathogen-specific transmission pathways and exposure settings identified can help inform prevention efforts.

Acute gastroenteritis (AGE; defined as diarrhea or vomiting) is a major cause of illness in the United States; an estimated 179 million episodes occur annually (*1*). AGE is caused by a variety of viral, bacterial, and parasitic pathogens and by toxins, chemicals, and other noninfectious causes. Noroviruses are the leading cause of epidemic gastroenteritis, detected in ≈50% of AGE outbreaks across Europe and the United States (*2*,*3*). However, until 2009, national surveillance for AGE outbreaks in the United States had been limited to foodborne or waterborne disease outbreaks because no national surveillance existed for AGE outbreaks spread by other transmission modes.

To better understand and guide appropriate interventions to prevent epidemic gastroenteritis, the Centers for Disease Control and Prevention (CDC) launched a novel national surveillance system in 2009—the National Outbreak Reporting System (NORS). This system enhanced and expanded upon 2 existing surveillance systems, the Foodborne Disease Outbreak Surveillance System and the Waterborne Disease and Outbreak Surveillance System. NORS is an Internet-based system for local, state, and territorial health departments to report all outbreaks of foodborne and waterborne disease; AGE outbreaks caused by contact with infected persons, animals, or environmental sources; and AGE outbreaks caused by other or unknown modes of transmission (*4*). As such, NORS provides a national surveillance system for all pathways of AGE outbreaks in the United States. To assess the roles of specific pathogens, temporal trends, and exposure pathways, we summarized AGE outbreak data submitted through NORS during the first 2 years after implementation of the system.

## The Study

In the United States, outbreaks (defined as >2 cases of a similar illness epidemiologically linked to a common exposure, e.g., setting or food) can be reported through NORS by all 50 US states, the District of Columbia, US territories (American Samoa, Guam, Commonwealth of the Northern Mariana Islands, Puerto Rico, and the United States Virgin Islands), and Freely Associated States (Federated States of Micronesia, Republic of Marshall Islands, and Republic of Palau). NORS was launched in February 2009, but sites were encouraged to report outbreaks that occurred since January 1, 2009. 

For analysis, we extracted data reported through NORS for AGE outbreaks in which the symptom onset date for the first reported illness was during January 1, 2009–December 31, 2010. Outbreaks of diseases that do not typically cause AGE (e.g., listeriosis, legionellosis, hepatitis A) were excluded from analysis (*1*). We analyzed various outbreak characteristics: date of first illness onset, primary transmission mode, confirmed or suspected etiology (*5*), exposure setting, and number of outbreak-associated illnesses, hospitalizations, and deaths. Primary mode of transmission is determined by each reporting site on the basis of the local public health investigation and CDC guidance documents (*6*).

Of 4,455 outbreaks reported through NORS during 2009–2010, a total of 4,376 (98%) were AGE outbreaks (1,883 in 2009, 2,493 in 2010) (Table 1), associated with 122,488 reported illnesses, 2,952 hospitalizations, and 168 deaths. A single suspected or confirmed etiology was implicated in 2,819 (64%) outbreaks, associated with 88,958 (73%) illnesses, 2,381 (81%) hospitalizations, and 146 (87%) deaths. Norovirus, the leading cause of single-etiology outbreaks, was responsible for 1,908 (68%) outbreaks, associated with 69,145 (78%) illnesses, 1,093 (46%) hospitalizations, and 125 (86%) deaths. *Salmonella *spp., *Shigella* spp*.*, and Shiga toxin–producing *Escherichia coli* (STEC), the next most frequently reported etiologic agents, were responsible for 355 (13%), 109 (4%), and 101 (4%) outbreaks, respectively. *Salmonella* spp. were the second most frequent cause of outbreak-associated hospitalizations (773 [32%]), and STEC was the second most frequent cause of outbreak-associated deaths (9 [6%]).

**Table 1:** Numbers of acute gastroenteritis outbreaks and outbreak-associated outcomes caused by various etiologic agents reported in the National Outbreak Reporting System, United States, 2009–2010*

Outbreak etiology	No. (%) outbreaks					No. (%) outbreak-associated outcomes		
	Confirmed		Suspected	Total		Illnesses	Hospitalizations	Deaths
Single agent†								
Norovirus‡	1,355 (64.2)		553 (78.1)	1,908 (67.7)		69,145 (77.7)	1,093 (45.9)	125 (85.6)
*Salmonella* spp.	344 (16.3)		11 (1.6)	355 (12.6)		8,590 (9.7)	773 (32.5)	6 (4.1)
*Shigella* spp.§	99 (4.7)		10 (1.4)	109 (3.9)		2,135 (2.4)	115 (4.8)	1 (0.7)
STEC	88 (4.2)		13 (1.8)	101 (3.6)		1,091 (1.2)	250 (10.5)	9 (6.2)
*Campylobacter* spp.¶	56 (2.7)		13 (1.8)	69 (2.4)		1,550 (1.7)	52 (2.2)	0
*Clostridium *spp.#	41 (1.9)		21 (3.0)	62 (2.2)		3,242 (3.6)	16 (0.7)	3 (2.1)
*Cryptosporidium* spp.**	17 (0.8)		30 (4.2)	47 (1.7)		598 (0.7)	21 (0.9)	1 (0.7)
*Bacillus* spp.††	13 (0.6)		12 (1.7)	25 (0.9)		522 (0.6)	3 (0.1)	0
*Staphylococcus aureus*	11 (0.5)		11 (1.6)	22 (0.8)		263 (0.3)	0	0
*Giardia intestinalis*	13 (0.6)		6 (0.8)	19 (0.7)		121 (0.1)	5 (0.2)	0
Sc ombroid toxin/histamine	18 (0.9)		0	18 (0.6)		76 (0.1)	0	0
Ciguatoxin	14 (0.7)		0	14 (0.5)		59 (0.1)	6 (0.3)	0
Rotavirus	9 (0.4)		5 (0.7)	14 (0.5)		372 (0.4)	9 (0.4)	0
Other‡‡	33 (1.6)		23 (3.2)	56 (2.0)		1,194 (1.3)	38 (1.6)	1 (0.7)
All single-agent etiologies	2,111 (98.9)		708 (31.6)	2,819 (64.4)		88,958 (72.6)	2,381 (80.7)	146 (86.9)
Multiple agents	24 (1.1)		9 (0.4)	33 (0.8)		1,236 (1.0)	61 (2.1)	2 (1.2)
Unknown agent	0		1,524 (68.0)	1,524 (34.8)		32,294 (26.4)	510 (17.3)	20 (11.9)
All outbreaks	2,135 (100.0)	2,241 (100.0)		4,376 (100.0)		122,488 (100.0)	2,952 (100.0)	168 (100.0)

*STEC, Shiga toxin–producing *Escherichia coli*. †Percentages for specific single agents are those among all single-agent etiology outbreaks (N = 2,819). ‡A norovirus genogroup was provided for 1,160 outbreaks: 150 GI, 1,003 GII, and 7 GI/GII.  §*S. sonnei* (95 confirmed and 8 suspected outbreaks), *S. flexneri* (5 confirmed outbreaks), *Shigella* sp. not known (1 confirmed outbreak). ¶*C. jejuni* (55 confirmed and 4 suspected outbreaks), *Campylobacter* sp. not known (8 confirmed and 2 suspected outbreaks). #*C. perfringens* (37 confirmed and 20 suspected outbreaks), *Clostridium* sp. not known (4 confirmed and 1 suspected outbreak). **C. parvum (10 confirmed and 1 suspected outbreak), *C. hominis* (6 confirmed outbreaks), *Cryptosporidium* sp. not known (30 confirmed outbreaks). ††*B. cereus* (13 confirmed and 11 suspected outbreaks), *Bacilllus* sp. not known (1 suspected outbreak). ‡‡Includes *Vibrio *sp. (8 outbreaks), cyanobacterial toxins (6 outbreaks), enterotoxigenic and enteropathogenic *E. coli* (4 outbreaks), *Enterococcus* spp. (3 outbreaks), mycotoxins (3 outbreaks), *Cyclospora* spp. (2 outbreaks), pesticides (2 outbreaks), sapovirus (2 outbreaks), paralytic shellfish poison (1 outbreak), *Pseudomonas* sp. (1 outbreak), sodium hydroxide (1 outbreak),* Yersinia* sp. (1 outbreak), and other unspecified etiologies (22 outbreaks).

**Figure 1:**
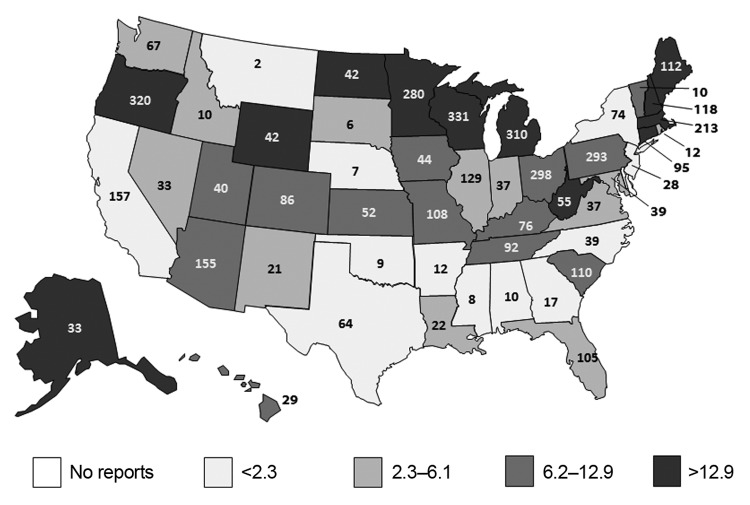
Total number and annual rate of reported acute gastroenteritis outbreaks per 1 million population by reporting state, National Outbreak Reporting System, United States, 2009–2010. The number given in each state indicates the total number of outbreaks over the 2-year study period; the shading denoted by the legend indicates the reporting rate by quartiles. Multistate outbreaks (n = 48) and those reported by Puerto Rico (n = 15) and the District of Columbia (n = 24) are not shown.

**Figure 2:**
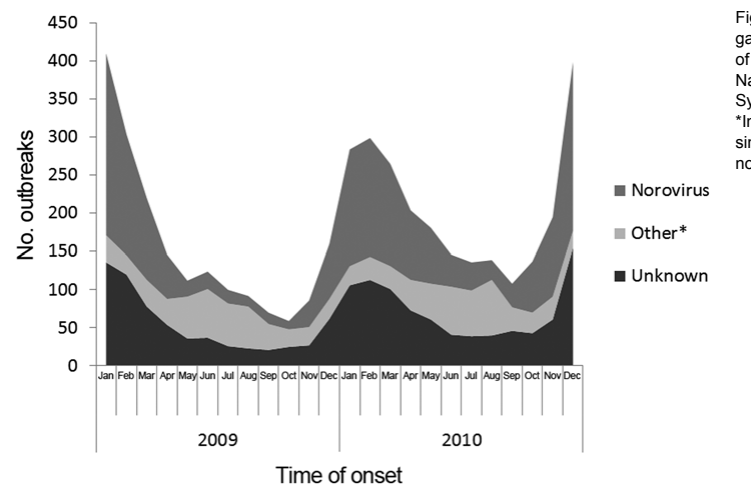
Number of reported acute gastroenteritis outbreaks by month of first illness onset and etiology, National Outbreak Reporting System, United States, 2009–2010. *Includes outbreaks caused by a single etiologic agent other than norovirus or multiple etiologies.

AGE outbreaks were reported by the District of Columbia, Puerto Rico, and all states except Delaware (Figure 1). A median of 42 outbreaks (range 2–331) was reported by each site, and the median rate was 7.3 outbreaks/1 million person-years (range 0.9–44.8). Overall, AGE outbreaks exhibited winter seasonality: 2,972 (68%) of the 4,376 outbreaks occurred during November–April (Figure 2). This trend was driven largely by outbreaks caused by norovirus and by unknown etiologies, of which 1,530 (80% of 1,908 total) and 1,086 (71% of 1,524 total), respectively, occurred during November–April. In contrast, 62% of outbreaks caused by other etiologies, primarily bacteria, occurred during May–October.

The primary reported mode of transmission in most AGE outbreaks was person to person (2,271 [52%]), followed by foodborne (1,513 [35%]), waterborne (65 [2%]), animal contact (44, 1%), and environmental contamination (9, 0.2%); the transmission mode was unknown in 474 (10%) outbreaks (Table 2). Person-to-person transmission was implicated in most outbreaks caused by norovirus (1,261 [66%]) and *Shigella* spp. (86 [79%]), whereas foodborne transmission was implicated in most outbreaks caused by *Salmonella* spp. (254 [72%]) and STEC (64 [63%]). Among the 3,052 (70%) AGE outbreaks for which a single exposure setting was reported, health care facilities, primarily nursing homes, were the most frequent settings (1,499 [49%]), followed by restaurants or banquet facilities (657 [22%]), schools or day-care facilities (290 [10%]), and private residences (227 [7%]). Most norovirus outbreaks (64%) occurred in health care facilities, whereas shigellosis outbreaks (74%) occurred predominantly in schools or day-care facilities. Private residences and restaurants/banquet facilities were the most frequent exposure settings for outbreaks caused by *Salmonella* spp. (32% and 36%, respectively) and STEC (46% and 20%, respectively).

**Table 2:** Primary transmission mode and exposure setting of acute gastroenteritis outbreaks, by etiologic agent, National Outbreak Reporting System, United States, 2009–2010*

Outbreak characteristic	No. (%) outbreaks						
	Norovirus, n = 1,908	*Salmonella* spp., n = 355	*Shigella* spp., n = 109	STEC, n = 101	Other, n = 379†	Unknown, n = 1,524	Total, N = 4,376
Primary transmission mode							
Person to person	1,261 (66.1)	17 (4.8)	86 (78.9)	11 (10.9)	47 (12.4)	849 (55.7)	2,271 (51.9)
Foodborne	494 (25.9)	254 (71.5)	8 (7.3)	64 (63.4)	220 (58.0)	473 (31.0)	1,513 (34.6)
Waterborne	4 (0.2)	0	2 (1.8)	6 (5.9)	51 (13.5)	2 (0.1)	65 (1.5)
Animal contact	0	26 (7.3)	0	5 (5.0)	12 (3.2)	1 (0.1)	44 (1.0)
Environmental contamination	5 (0.3)	2 (0.6)	1 (0.9)	0	0	1 (0.1)	9 (0.2)
Unknown	144 (7.5)	56 (15.8)	12 (11.0)	15 (14.9)	49 (12.9)	198 (13.0)	474 (10.8)
Exposure setting‡							
Health care facility	932 (48.8)	5 (1.4)	0	0	25 (6.6)	537 (35.2)	1,499 (34.3)
Restaurant or banquet facility	287 (15.0)	69 (19.4)	5 (4.6)	12 (11.9)	77 (20.3)	207 (13.6)	657 (15.0)
School or day-care facility	98 (5.1)	14 (3.9)	50 (45.9)	6 (5.9)	15 (4.0)	107 (7.0)	290 (6.6)
Private residence	31 (1.6)	62 (17.5)	4 (3.7)	28 (27.7)	60 (15.8)	42 (2.8)	227 (5.2)
Other single setting	114 (6.0)	42 (11.8)	9 (8.3)	15 (14.9)	101 (26.6)	98 (6.4)	379 (8.7)
Multiple	33 (1.7)	19 (5.4)	13 (11.9)	10 (9.9)	10 (2.6)	21 (1.4)	106 (2.4)
Not reported	264 (13.8)	86 (24.2)	15 (13.8)	15 (14.9)	42 (11.8)	313 (20.5)	735 (16.8)
Not collected§	149 (7.8)	58 (16.3)	13 (11.9)	15 (14.9)	49 (12.9)	199 (13.1)	483 (11.0)

*Data include both suspected and confirmed etiologies. STEC, Shiga toxin-producing *Escherichia coli*.  †Includes outbreaks caused by a single etiologic agent other than norovirus, *Salmonella* spp., *Shigella* spp., and STEC or by multiple etiologic agents, as listed in Table 1. ‡Data on specific settings are restricted to outbreaks with a single exposure setting; for foodborne outbreaks, setting refers to the setting where implicated food was consumed. §The setting was systematically not collected for outbreaks caused by environmental contamination or unknown transmission mode.

## Conclusions

As the national surveillance system for US AGE outbreaks, NORS provides valuable insights into the epidemiology of the pathogens most often involved. Building upon previous surveillance systems and analyses focused on specific transmission modes (*7*–*10*), NORS provides a more complete characterization of AGE outbreaks, particularly the relative importance of specific transmission modes and settings for the key pathogens. This analysis highlights norovirus as not only the leading cause of reported AGE outbreaks but also the leading cause of AGE outbreak–associated hospitalizations and deaths. Although norovirus usually causes self-limiting disease, it can cause severe outcomes when outbreaks occur among vulnerable populations, such as nursing-home residents (*11*). *Salmonella* spp*.*, *Shigella* spp., and STEC are also key contributors to AGE outbreaks. Expanded surveillance through NORS revealed that 28%, 91%, and 31%, respectively, of outbreaks caused by these 3 bacteria result from routes other than contaminated food or water. In addition, NORS provides information on non-AGE outbreaks transmitted by food or water. For example, ≈25% of waterborne disease outbreaks are caused by *Legionella* spp. (*8*,*9*), and among foodborne disease outbreaks, listeriosis is a major cause of outbreak-related hospitalizations and deaths (*10*).

As a passive reporting system, NORS is subject to variability in reporting practices between states and among outbreaks associated with different transmission modes and exposure settings. Reporting rates and data completeness may be improved through ongoing NORS enhancements, including direct data upload functionality and all-mode collection of setting information. For 35% of outbreaks, no suspected or confirmed etiology was identified, primarily because diagnostic specimens were not collected. However, outbreaks of unknown etiology exhibited similar temporal trends and epidemiologic characteristics as norovirus outbreaks, suggesting that many of these may have been caused by norovirus. NORS does not include AGE outbreaks on international cruise ships; however, if combined with the outbreaks reported to NORS, these cruise-ship outbreaks would represent <1% of all reported outbreaks attributed to norovirus and all-cause AGE (*12*).

Although a small minority of AGE cases in the United States are associated with reported outbreaks (≈1 in 3,000), outbreak surveillance provides unique insights that can inform prevention efforts. Norovirus control through hand hygiene, environmental disinfection, and isolation of ill persons should remain a priority and likely affords protection against other AGE agents (*13*). Ongoing surveillance through NORS will help further elucidate trends, identify gaps, and assess the effects of future interventions on reducing epidemic gastroenteritis.
